# The novel bio-SYNTAX scoring system for predicting the prognosis of patients undergoing percutaneous coronary intervention with left main coronary artery disease

**DOI:** 10.3389/fcvm.2022.912286

**Published:** 2022-09-23

**Authors:** Jae Yong Yoon, Jang Hoon Lee, Hong Nyun Kim, Namkyun Kim, Se Yong Jang, Myung Hwan Bae, Dong Heon Yang, Hun Sik Park, Yongkeun Cho

**Affiliations:** ^1^Department of Internal Medicine, CHA Gumi Medical Center, CHA University, Gumi, South Korea; ^2^Department of Internal Medicine, Kyungpook National University Hospital, Daegu, South Korea; ^3^School of Medicine, Kyungpook National University, Daegu, South Korea; ^4^Department of Internal Medicine, Kyungpook National University Chilgok Hospital, Daegu, South Korea

**Keywords:** risk stratification, N-terminal pro-B type natriuretic peptide, left main coronary artery disease, percutaneous coronary intervention, drug eluting stent

## Abstract

**Background:**

Simple and effective risk models incorporating biomarkers associated with left main coronary artery (LMCA) stenosis are limited. This study aimed to validate the novel Bio-Clinical SYNTAX score (Bio-CSS) incorporating N-terminal pro-B-type natriuretic peptide (NT-proBNP) in patients with LMCA stenosis.

**Methods:**

Patients who underwent percutaneous coronary intervention (PCI) for LMCA stenosis using a drug-eluting stent (*n* = 275) were included in the study. We developed the Bio-CSS incorporating NT-proBNP and validated the ability of the Bio-CSS to predict major adverse cardiac events (MACEs) and compared its performance to that of the SYNTAX score (SS) and SS II. The MACEs were defined as death, non-fatal myocardial infarction (MI), and repeat revascularizations.

**Results:**

The Bio-CSS (34.7 ± 18.3 vs. 51.9 ± 28.4, *p* < 0.001), as well as SS (23.6 ± 7.3 vs. 26.7 ± 8.1, *p* = 0.003) and SS II (29.4 ± 9.9 vs. 36.1 ± 12.8, *p* < 0.001), was significantly higher in patients with MACEs. In the Cox proportional hazards model, the log Bio-CSS (hazard ratio 8.31, 95% CI 1.84–37.55) was an independent prognostic factor for MACEs after adjusting for confounding variables. In the receiver operating characteristic curves, the area under the curve of the Bio-CSS was significantly higher compared to those of SS (0.608 vs. 0.706, *p* = 0.001) and SS II (0.655 vs. 0.706, *p* = 0.026). Patients were categorized into the three groups based on the tertiles of the Bio-CSS. Patients in the highest tertile of the Bio-CSS had significantly higher MACEs compared to those in the lower two tertiles (log-rank *p* < 0.001).

**Conclusion:**

In patients who underwent PCI for LMCA stenosis, the novel Bio-CSS improved the discrimination accuracy of established combined scores, such as SS and SS II. The addition of NT-proBNP to the clinical and angiographic findings in the Bio-CSS could potentially provide useful long-term prognostic information in these patients.

## Introduction

The advances in percutaneous coronary intervention (PCI) techniques have improved the clinical outcomes of unprotected left main coronary artery (LMCA) stenosis (1–5). However, it is still uncertain whether PCI with the current drug-eluting stent (DES) is non-inferior to coronary artery bypass graft (CABG) surgery for a clinical outcome or not (6, 7). Therefore, risk stratification is crucial for the improvement of clinical outcomes in patients with LMCA stenosis undergoing PCI. The Synergy between PCI with Taxus and Cardiac Surgery (SYNTAX) score (SS) system was developed to predict the risk of major adverse cardiac events (MACEs) after PCI (8–10). However, the ability of SS to ascertain 1-year MACEs was insufficient for patients with LMCA stenosis who underwent PCI due to insufficient clinical information. Therefore, effective risk models, which improve the performance of SS in these patient subsets, are essential.

Biomarkers such as N-terminal pro-B-type natriuretic peptide (NT-proBNP) could provide useful prognostic information in patients with coronary artery disease (11–13). However, simple and effective risk models incorporating relevant biomarkers in patients with LMCA are limited. Therefore, we developed the Bio-Clinical SS (Bio-CSS), which incorporates NT-proBNP and validated the ability of the Bio-CSS to predict MACEs, especially compared to that of SS and SS II in patients with LMCA stenosis who underwent PCI.

## Materials and methods

### Study design and patient population

**Figure 1:**
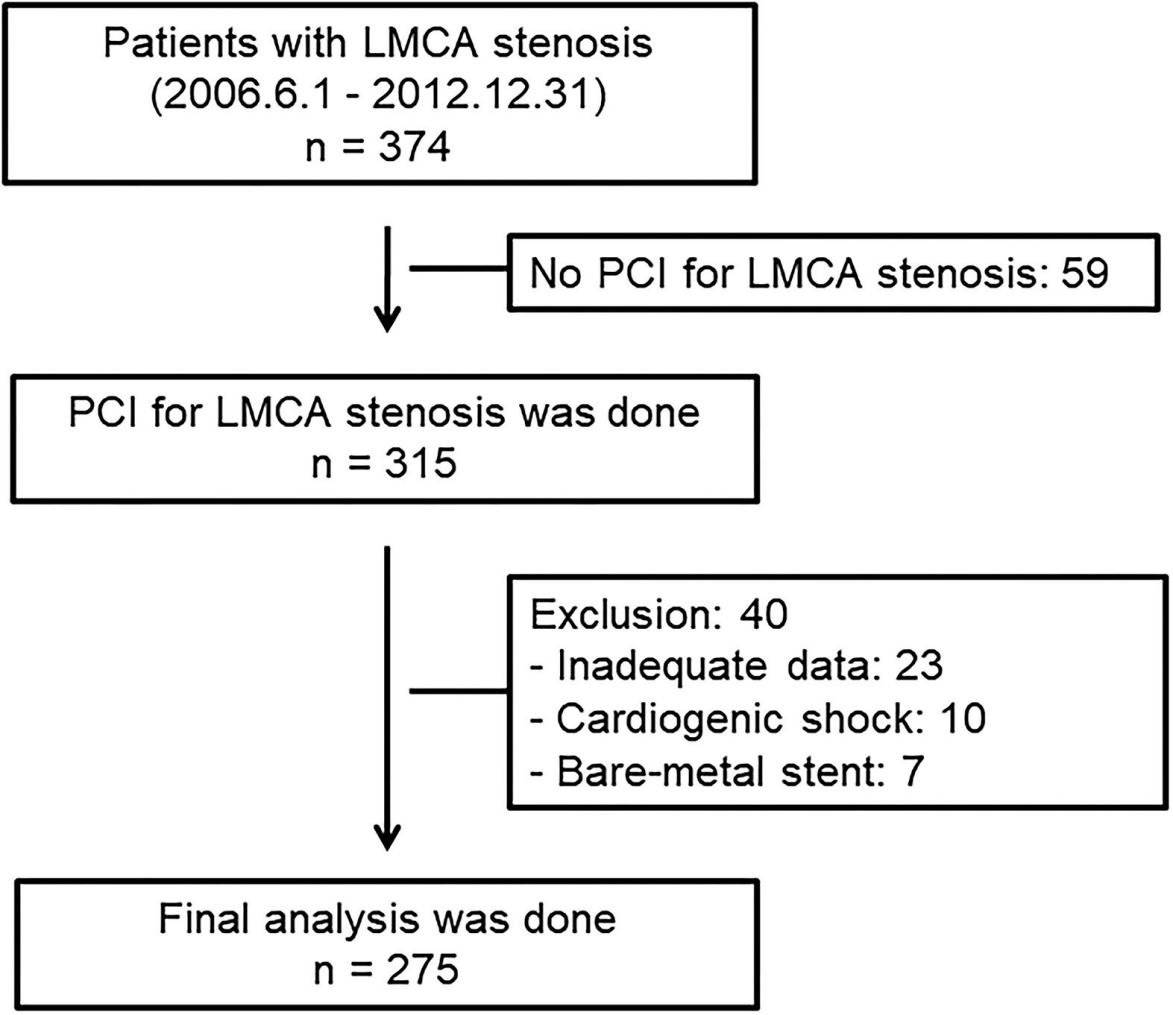
Flowchart of the study patients. LMCA, left main coronary artery; PCI, percutaneous coronary intervention.

This observational study included 374 consecutive patients with *de-novo* unprotected LMCA stenosis who were admitted for coronary angiography between June 2006 and December 2012. Patients with significant *de-novo* unprotected LMCA stenosis were enrolled in this study. Significant unprotected LMCA stenosis was defined as severe LMCA diameter stenosis (>70%) as determined by angiography, or intermediate LMCA stenosis (50–69%) as determined by angiography with intravascular ultrasound (IVUS)-derived minimal luminal area of < 6 mm^2^. Patients with cardiogenic shock, cardiac arrest during hospitalization, protected LMCA stenosis, and bare-metal stent were excluded from this study. The choice of revascularization modality was mainly determined by attending physicians based on contemporary guidelines. As a rule, patients with significant LMCA stenosis and complex anatomy were recommended CABG as the first revascularization modality. If they declined CABG, PCI was performed as an alternative therapy. PCI was performed for LMCA stenosis in 315 patients. Overall, 40 patients were excluded from this study, including 23 patients with inadequate data, 10 patients with cardiogenic shock, and 7 patients with bare-metal stent implantation. Finally, 275 patients who underwent PCI for LMCA stenosis with DES were analyzed in this study. The flowchart of the study is given in Figure 1. The study protocols were approved by the Institutional Review Boards of Kyungpook National University Hospital (No. KNUH 2020-06-006). Informed consent was waived by the board.

We analyzed the baseline demographic and clinical characteristics, including age, sex, cardiovascular risk factors (hypertension, diabetes mellitus, hyperlipidemia, and family history of coronary heart disease), and comorbidities. ECG was recorded and analyzed in all the patients by attending cardiologists. Venous blood specimens were obtained on admission. The serum creatinine was determined using standard methods. The NT-proBNP level was quantified using an electrochemiluminescence immunoassay method (Modular Analytics E170, Roche Diagnostics, Germany). The left ventricular ejection fraction (LVEF) was determined using two-dimensional echocardiography at the index hospitalization.

Standard interventional techniques were used for all the procedures. The main treatment principles of the PCI procedure were as follows: wiring of the LMCA to the left anterior descending (LAD) and/or left circumflex (LCX) artery, predilatation of stenosed areas of the LMCA before IVUS examination if the passage of IVUS catheter is not possible, IVUS examination at the operator's discretion, implantation of the stent from LMCA to LAD or LCx, postdilatation with the single or final kissing balloon technique at the operator's discretion, and IVUS examination after stenting. The IVUS images were obtained using a manual or automatic fullback system *via* commercially available imaging systems (40 MHz IVUS catheter, Boston Scientific: 20 MHz IVUS catheter, Volcano, Rancho Cordova, California, USA). A preinterventional IVUS examination provided information about the characterization of plaque and guided treatment strategy, including the selection of appropriate diameter for balloons and stents. The poststenting IVUS examination enables the evaluation of stent expansion and apposition and aids in deciding on additional procedures.

Antiplatelet therapy and periprocedural anticoagulation were performed using standardized regimens. Before the procedure, all the patients received a loading dose of aspirin (300 mg) and clopidogrel (300 or 600 mg). In the catheterization laboratory, a bolus of unfractionated heparin (75–100 U/kg) was administered for anticoagulation, to achieve an activated clotting time > 300 s. The routine use of postprocedure unfractionated heparin was not recommended unless the patients required intra-aortic balloon pumps. The use of glycoprotein IIb/IIIa receptor inhibitors was left to the attending interventional cardiologist's judgment. Postprocedure, the patients were prescribed aspirin (100 mg) and clopidogrel (75 mg) for at least 12 months, potentially longer, based on the operator's discretion.

### Bio-clinical synergy between PCI with taxus and cardiac surgery score

The SS and SS II scores for each patient were calculated by scoring all the coronary lesions with diameter stenosis ≥ 50%, in vessels ≥ 1.5 mm, using the SS algorithm and are available on the SS website (www.syntaxscore.com) (8, 9). The age, creatinine, and ejection fraction (ACEF) score was calculated using the following formula: ACEF = Age/LVEF + 1 (if creatinine was > 2.0 mg/dl) (14). The clinical SS (CSS) was calculated retrospectively for every patient using the formula CSS = (SS) × (ACEF score). The Bio-CSS was calculated by adding the log-transformed NT-proBNP levels to CSS (CSS + log NT-proBNP).

### Clinical outcomes

The mean follow-up duration was 1,625 ± 931 days. The patients were followed-up for more than 1 year. The MACEs were defined as death, non-fatal myocardial infarction, and repeat revascularization, including PCI and CABG. During the follow-up period, the follow-up data were obtained by reviewing medical records and telephone interviews with patients.

### Statistical analyses

Data are expressed as mean ± SD for continuous variables and percentages for categorical variables. All the comparisons between the baseline variables were assessed using Student's *t*-test for continuous variables and Pearson's chi-squared test for categorical variables. The patients were categorized into the three groups based on the tertiles of the Bio-CSS: Bio-CSS_LOW_ < 28 (*n* = 84), 28 ≤ Bio-CSS_MID_ < 39 (*n* = 95), and Bio-CSS_HIGH_ ≥ 39 (*n* = 96). The cumulative incidence rates of MACE and the mortality based on the Bio-CSS tertiles were estimated using the Kaplan–Meier method, and further compared by using the log-rank test. Univariate analyses were performed to determine the predictors for MACEs. The Cox proportional-hazards model was used to calculate the hazard ratios (HRs) and the CIs for the independent predictors of MACEs. The variables with *p*-values ≤ 0.05 on the univariate analysis were entered into the Cox proportional-hazards model. The Hosmer–Lemeshow chi-square—a measure of deviation between the observed and predicted outcomes in deciles of predicted risk—was used to evaluate the calibration of the model.

The increased discriminative value of the Bio-CSS compared to the SS and SS II was estimated using three measures (Harrell's C-index, net reclassification improvement, and integrated discrimination improvement). Harrell's C-index (c-statistic) was defined as the proportion of usable patient pairs, in which the predictions and outcomes were concordant (1). We estimated the receiver operating characteristic (ROC) curves and compared the areas under the ROC curves (AUC) for the SS, SS II, and Bio-CSS in corresponding logistic models (15). The net reclassification improvement and integrated discrimination improvement were calculated by analyzing the differences in the individual estimated probabilities for MACEs of the Bio-CSS to SS and SS II (16). Because no prior risk categories exist for MACEs, we calculated the category-free net reclassification improvement (16). For all the analyses, a two-sided *p* < 0.05 was considered statistically significant. The statistical analysis was performed using SAS software (version 9.3, SAS Institute, Cary, North Carolina, USA).

## Results

The mean age of the participants was 64.5 ± 10.6 years, and 204 (74.2%) were men. The mean Bio-CSS was 39.7 ± 23.0 (median, 33.0; range, 12.1–182.3). The baseline characteristics of the study population are shown in Table 1. The age, prevalence of acute coronary syndrome, serum levels of creatinine, and NT-proBNP significantly increased as the Bio-CSS tertile increased, whereas the LVEF significantly decreased as the Bio-CSS tertile increased. The indicators of lesion complexity, such as the number of diseased vessels, presence of left main (LM) bifurcation, and small vessels with the long lesions, were significantly higher in the Bio-CSS_HIGH_ tertile compared to the other groups (Table 2).

**Table 1:** Clinical characteristics of the study subjects.

**Variables**	**Bio-CSS < 28 (*N =* 84)**	**28 ≤ Bio-CSS < 39** **(*N =* 95)**	**Bio-CSS ≥ 39 (*N =* 96)**	***p* value**
Bio-CSS	22.1 ± 4.4	32.7 ± 3.4	61.9 ± 26.1	< 0.001
Age (year)	57.0 ± 10.8	65.4 ± 7.8	70.2 ± 8.7	< 0.001
Male, *n* (%)	61 (72.6)	74 (77.9)	69 (71.9)	0.589
Body mass index (Kg/m^2^)	23.9 ± 2.3	23.9 ± 2.7	22.9 ± 2.7	0.058
Clinical presentation				< 0.001
Chronic stable angina, *n* (%)	26 (31.0)	38 (40.0)	11 (11.5)	
Acute coronary syndrome, *n* (%)	58 (69.0)	57 (60.0)	85 (88.5)	
Medical history				
Coronary heart disease, *n* (%)	12 (14.8)	18 (20.5)	24 (27.3)	0.137
Hypertension, *n* (%)	35 (43.2)	54 (61.4)	48 (54.5)	0.059
Diabetes mellitus, *n* (%)	20 (24.7)	31 (35.2)	36 (40.9)	0.079
Hyperlipidemia, *n* (%)	22 (27.2)	34 (38.6)	24 (27.3)	0.172
Current smoker, *n* (%)	48 (59.3)	54 (61.4)	54 (61.4)	0.950
Left ventricular ejection fraction (%)	58.9 ± 7.1	57.7 ± 7.5	45.0 ± 13.1	< 0.001
Serum creatinine (mg/dL)	0.80 ± 0.22	1.04 ± 0.78	1.45 ± 1.52	< 0.001
Log NT-proBNP (pg/mL)	4.7 ± 1.2	5.5 ± 1.3	7.0 ± 1.7	< 0.001
Discharge medication				
Aspirin, *n* (%)	83 (98.8)	95 (100.0)	95 (99.0)	0.584
Clopidogrel, *n* (%)	81 (96.4)	94 (98.9)	94 (97.9)	0.514
ACE-I/ARBs, *n* (%)	71 (84.5)	75 (78.9)	70 (72.9)	0.166
Beta-blockers, *n* (%)	74 (88.1)	88 (92.6)	78 (81.2)	0.06
Statins, *n* (%)	68 (81.0)	70 (73.7)	74 (77.1)	0.513
Diuretics, *n* (%)	6 (7.1)	16 (16.8)	34 (35.4)	< 0.001

Data expressed as mean ± SD or number (percent).

SS, SYNTAX score; SS II, SYNTAX score II; Bio-CSS, Biomarker-Clinical SYNTAX score; NT-proBNP, N-terminal pro-B type natriuretic peptide; ACE-I/ARBs, Angiotensin-converting enzyme inhibitors/angiotensinogen type II receptor blockers.

**Table 2:** Angiographic and procedural characteristics of the study subjects.

**Variables**	**Bio-CSS < 28 (*N =* 84)**	**28 ≤ Bio-CSS < 39** **(*N =* 95)**	**Bio-CSS≥39 (*N =* 96)**	***p* value**
LMCA status				< 0.001
LMCA, isolated, *n* (%)	24 (28.6%)	1 (1.1%)	3 (3.1%)	
LMCA + 1-vessel disease, *n* (%)	17 (20.2%)	15 (15.8%)	5 (5.2%)	
LMCA + 2-vessel disease, *n* (%)	16 (19.0%)	31 (32.6%)	15 (15.6%)	
LMCA + 3-vessel disease, *n* (%)	27 (19.8%)	48 (50.5%)	73 (76.0%)	
LM bifurcation	55 (65.5%)	82 (86.3%)	85 (88.5%)	< 0.001
LM Stent size (mm)	3.56 ± 0.34	3.57 ± 0.50	3.39 ± 0.32	0.003
LM Stent length (mm)	21.8 ± 5.95	23.98 ± 6.07	23.55 ± 6.29	0.049
Reference vessel diameter (mm)	3.58 ± 0.48	3.47 ± 0.41	3.40 ± 0.38	0.025
Minimal lumen diameter (mm)	1.73 ± 0.43	2.01 ± 2.45	1.73 ± 1.64	0.455
Drug-eluting stent type				0.815
Sirolimus eluting stent, *n* (%)	3 (3.6%)	5 (5.3%)	2 (2.1%)	
Paclitaxel eluting stent, *n* (%)	11 (13.1%)	14 (14.7%)	20 (20.8%)	
Zotarolimus eluting stent, *n* (%)	23 (27.4%)	24 (25.3%)	28 (29.2%)	
Everolimus eluting stent, *n* (%)	38 (45.2%)	45 (47.3%)	40 (41.7%)	
Biolimus eluting stent, *n* (%)	9 (10.7%)	7 (7.4%)	6 (6.2%)	
LM stenting strategy				0.981
1 stent strategy, *n* (%)	75 (89.3%)	84 (88.4%)	85 (88.5%)	
2 stent strategy, *n* (%)	9 (10.7%)	11 (11.6%)	11 (11.5%)	

Data expressed as mean ± SD or number (percent).

LMCA, left main coronary artery; LM, left main.

During the follow-up, 80 (29.1%) MACEs, including 55 (20%) all-cause deaths, 23 (8.4%) non-fatal MIs, and 16 (5.8%) revascularizations, occurred (Table 3). Overall, the MACEs (49.0 Bio-CSS_HIGH_ vs. 23.2 Bio-CSS_MID_ vs. 13.1% Bio-CSS_LOW_, *p* < 0.001) and mortality (41.7 Bio-CSS_HIGH_ vs. 12.6 Bio-CSS_MID_ vs. 3.6% Bio-CSS_LOW_, *p* < 0.001) were significantly higher in the Bio-CSS_HIGH_ tertile as compared to the two lower tertiles.

**Table 3:** Clinical outcomes during the follow-up.

**Variables**	**Bio-CSS < 28 (*N =* 84)**	**28 ≤ Bio-CSS < 39** **(*N =* 95)**	**Bio-CSS ≥ 39 (*N =* 96)**	***p* value**
Major adverse cardiac events, *n* (%)	11 (13.1)	22 (23.2)	47 (49.0)	< 0.001
Death, *n* (%)	3 (3.6)	12 (12.6)	40 (41.7)	< 0.001
Non-fatal MI, *n* (%)	5 (6.0)	6 (6.3)	12 (12.5)	0.192
Revascularizations, *n* (%)	5 (6.0)	6 (6.3)	5 (5.2)	0.946

Data expressed as number (percent).

Bio-CSS, Biomarker-Clinical SYNTAX score; MI, myocardial infarction.

**Figure 2:**
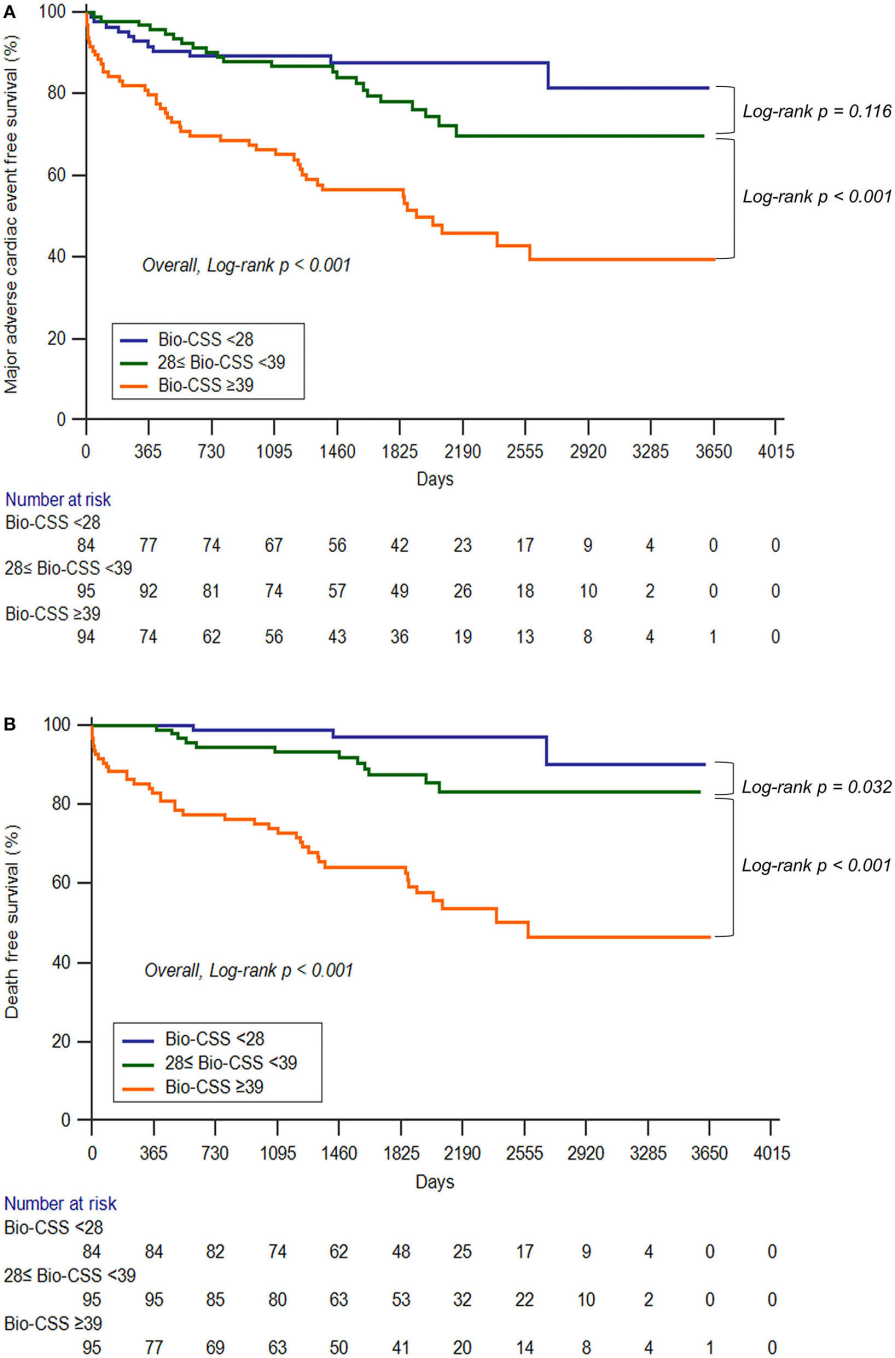
The Kaplan–Meier survival curves depict the major adverse cardiac events **(A)** and mortality **(B)** according to the Bio-CSS tertiles. Bio-CSS, Biomarker-clinical SYNTAX score.

In univariate analysis for MACEs, the Bio-CSS (34.6 ± 18.2 vs. 51.8 ± 28.3, *p* < 0.001), SS (23.6 ± 7.2 vs. 26.7 ± 8.0, *p* = 0.002), and SS II (29.4 ± 9.9 vs. 36.0 ± 12.8, *p* < 0.001) were significantly higher in patients with MACEs than in those patients without MACEs (Supplementary Table 1). The log-transformed NT-proBNP level was significantly higher in patients with MACEs than in those patients without MACEs (5.45 ± 1.52 vs. 6.51 ± 2.04, *p* < 0.001). Patients with MACEs were more likely to be male and had acute coronary syndromes. The use of beta-blockers and statins was significantly higher, and the use of diuretics was significantly lower in patients with MACEs. As per the Cox proportional-hazards model (Table 4), the log Bio-CSS (HR 8.31, 95% CI 1.84–37.55; *p* = 0.006) and statin therapy (HR 0.49, 95% CI 0.28–0.86; *p* = 0.012) were independent prognostic factors for MACEs after adjusting for confounding variables. The Kaplan–Meier survival curve analysis indicated that patients in the Bio-CSS_HIGH_ tertile had significantly higher rates of MACEs when compared with the lower 2 tertiles (log-rank *p* < 0.001; Figure 2A). Additionally, the mortality rate was significantly higher for the Bio-CSS_HIGH_ tertile compared to the lower two tertiles (log-rank *p* < 0.001; Figure 2B).

**Table 4:** Multivariate predictors of major adverse cardiac events during the follow-up.

**Variables**	**HR**	**95% CI**	***p* value**
Male	1.99	0.93–4.23	0.075
Acute coronary syndrome	1.27	0.67–2.39	0.460
Beta-blockers	0.69	0.37–1.31	0.256
Statins	0.49	0.28–0.86	0.012
Diuretics	1.44	0.78–2.66	0.239
Log Bio-CSS	8.31	1.84–37.55	0.006

HR, hazard ratio; CI, confidence interval; Bio-CSS, Biomarker-Clinical SYNTAX score.

**Figure 3:**
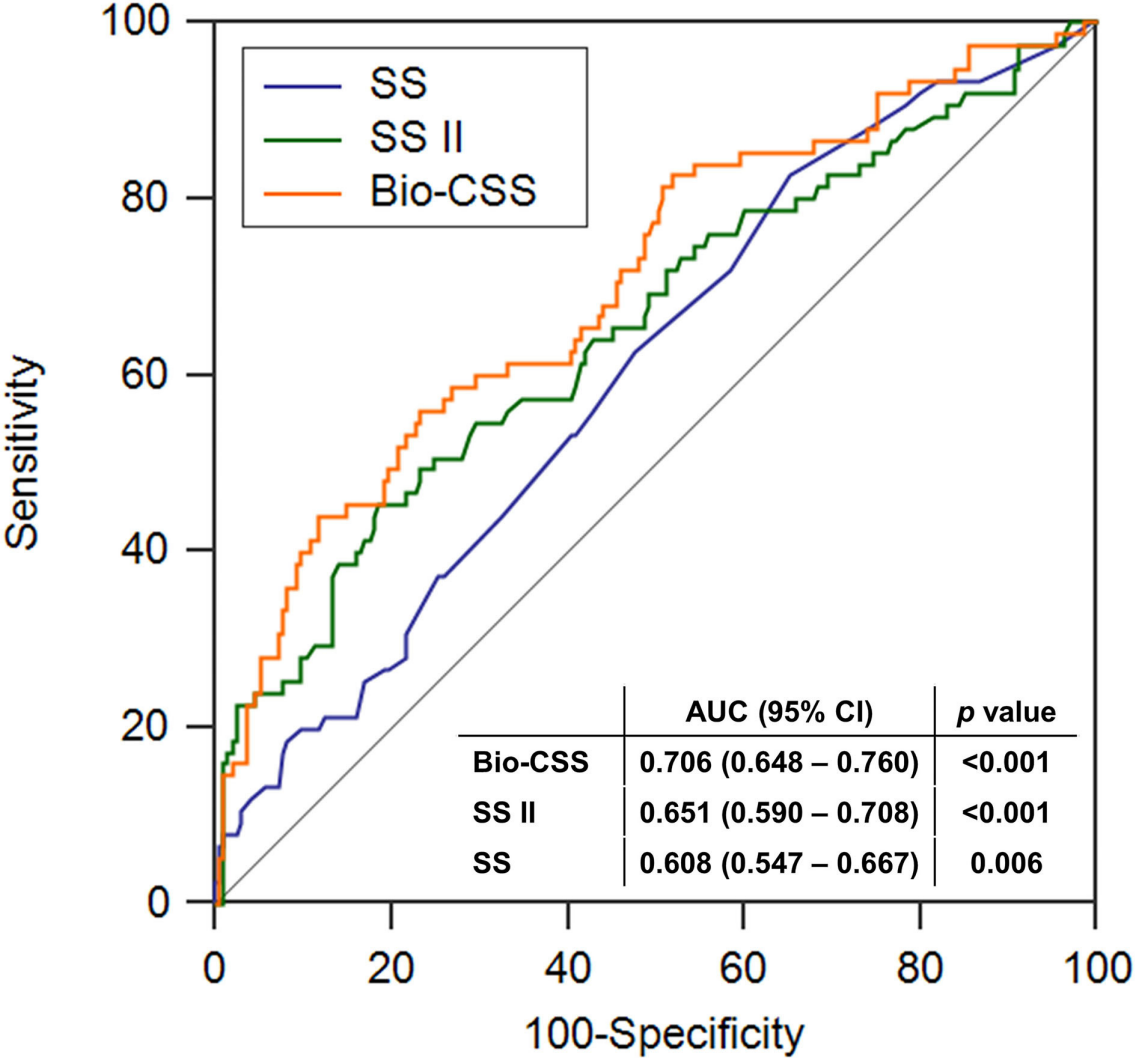
The receiver operating characteristics analysis of the SS, SS II, and Bio-CSS for major adverse cardiac events. SS, SYNTAX score; SS II, SYNTAX score II; Bio-CSS, Biomarker-clinical SYNTAX score; AUC, area under the curve; CI, confidence interval.

The AUC for the ROC analysis of the Bio-CSS for predicting MACEs was 0.706 (Figure 3) and significantly higher compared to SS (0.608, *p* = 0.001) and SS II (0.655, *p* = 0.026) (Table 5). The Bio-CSS significantly improved the reclassification (0.617; *p* < 0.001) and integrated discrimination (0.084; *p* < 0.001) of the patients compared to SS. No improvements were seen in SS II for the AUC for the prediction of MACEs of patients compared to SS (*p* = 0.345). The Bio-CSS also significantly improved the reclassification (0.273; *p* = 0.043) and integrated discrimination (0.045; *p* = 0.003) of the patients compared to SS II.

**Table 5:** Discrimination of the SYNTAX score, the SYNTAX score II, and the Bio-Clinical SYNTAX score in predicting major adverse cardiac events.

**Variables**	**Discrimination**					
	**C- index**	***p* value**	**NRI**	***p* value**	**IDI**	***p* value**
SS	0.608		Reference		Reference	
SS II	0.651	0.345	0.302	0.025	0.038	0.045
Bio-CSS	0.706	0.001	0.617	< 0.001	0.084	< 0.001
SS II	0.651		Reference		Reference	
Bio-CSS	0.706	0.026	0.273	0.043	0.045	0.003

SS, SYNTAX score; CSS, Clinical SYNTAX score; Bio-CSS, Biomarker-Clinical SYNTAX score; NRI, Net Reclassification Improvement; IDI, Integrated Discrimination Improvement.

The NRI was defined as (P_improved prediction among patients with major adverse cardiac events_ + P_improved prediction among patients without major adverse cardiac events_) (P_worsened prediction among_patients with major__adverse cardiac events_ + P_worsened prediction among patients without major adverse cardiac events_), where p = proportion of patients. The IDI was defined as ($\sum_{\text{major adverse cardiac events}}^{\text{i}}$ (P_new_(i) P_old_(i))/n (Patients with major adverse cardiac events)) ($\sum_{\text{no major adverse cardiac events}}^{\text{j}}$ (P_new_(j) P_old_(j))/n (Patients without major adverse cardiac events)), where p = predicted probability of major adverse cardiac events.

## Discussion

The main findings from this study are as follows. First, patients with the higher Bio-CSS have high-risk clinical and angiographic characteristics. Second, the novel Bio-CSS is an independent predictor of MACEs in patients who underwent PCI with LMCA stenosis. Third, the patients with the highest Bio-CSS tertiles have worse clinical outcomes. Fourth, the novel Bio-CSS was found to be superior to both the SS and SS II in the prediction of MACEs in patients who underwent PCI with LMCA stenosis.

There are two significant findings in our study. First, to the best of our knowledge, this is the first risk prediction model incorporating the NT-proBNP levels of patients who underwent PCI with LMCA stenosis. NT-proBNP is a well-known predictor of clinical outcomes in patients with coronary artery disease (17). The variables included in the CSS score—age, creatinine, and LVEF—are well-known contributors to the risk of LMCA stenosis (18, 19). In patients with chronic heart failure, the plasma levels of NT-proBNP are influenced by age, renal function, and LVEF (20–22). However, in patients with coronary artery disease, NT-proBNP was an independent predictor of all-cause mortality after adjustment for age and LVEF (17). Therefore, despite the close links among the NT-proBNP, age, creatinine level, and LVEF, the NT-proBNP can provide valuable additional prognostic information beyond the conventional risk factors.

Second, the Bio-CSS has a robust prognostic accuracy compared with the SS and SS II, and accurately stratifies the patients for long-term clinical outcomes in real-world patients who underwent PCI with LMCA stenosis. The original SS was developed based on coronary anatomy and lesion characteristics (9, 14). Although the SS was good at predicting the overall MACEs, the absence of any clinical characteristics in the SS calculation limited the scope for improvement of the predictive ability of risk scores in patients with LMCA stenosis (23). The SS II was developed to overcome these limitations. In the previous studies (DELTA and CREDO-Kyoto registry), the predictive ability of the SS II was superior for all-cause mortality compared to the anatomical SS in patients treated with PCI for LMCA stenosis and complex coronary artery disease (24, 25). However, it includes the two anatomical and six clinical factors for the prediction of 4-year mortality in the patients undergoing PCI or CABG. The incorporation of too many variables in the risk model—with the aim of creating an “optimal model”—may result in statistical overfitting and instability (26). A simple model may occasionally outperform a more complex model. The CSS is simple, practical, and easy to calculate by multiplying the SS with the ACEF score (using only the age, creatinine level, and LVEF) (10). Although the CSS had a better index of separation for most ischemic endpoints compared to the SS, the rate of MACEs was comparable between the SS and CSS in patients who underwent PCI with acute coronary syndrome (27). Therefore, in the previous study, we developed and validated the Bio-CSS for the first time to improve the prediction ability of the CSS for clinical outcomes in patients with acute myocardial infarction (28). Although the external validation of the Bio-CSS was not performed in the present study, we believe that the Bio-CSS could be applied to patients who underwent PCI with LMCA stenosis for the best risk prediction model.

This study has certain limitations. First, our study is not a randomized and controlled study. Therefore, we cannot completely exclude the possibility of residual confounding factors that were not available in our registry. Second, the ROC method of analysis may not be appropriate for the present study, as it is only suited for diagnostic purposes. Although the ROC method has not been extensively validated for prognostic models because these models must incorporate time-censored data (29), the same method has been used in the previously published study (28). Despite these limitations, we believe that the Bio-CSS could provide the necessary clinical insight to determine the prognosis of patients who underwent PCI with LMCA stenosis.

In conclusion, an improvement in the ability of the SS and SS II for the prediction of long-term MACEs can be achieved by combining the CSS with the NT-proBNP level to formulate the Bio-CSS. The Bio-CSS is a novel valid model for the prediction of long-term MACEs in patients undergoing PCI with LMCA stenosis.

## Data availability statement

The raw data supporting the conclusions of this article will be made available by the authors, without undue reservation.

## Ethics statement

The studies involving human participants were reviewed and approved by the Institutional Review Boards of Kyungpook National University Hospital (No. KNUH 2020-06-006). Written informed consent for participation was not required for this study in accordance with the national legislation and the institutional requirements.

## Author contributions

JY, JL, and HP contributed to the conception and design of this study. HK and NK conducted the investigations and organized the database. JL wrote the first draft of the manuscript. JY, MB, DY, and YC wrote sections of the manuscript. All authors have contributed to manuscript revision, reading, and approval of the submitted version of the manuscript.

## Conflict of interest

The authors declare that the research was conducted in the absence of any commercial or financial relationships that could be construed as a potential conflict of interest.

## Publisher's note

All claims expressed in this article are solely those of the authors and do not necessarily represent those of their affiliated organizations, or those of the publisher, the editors and the reviewers. Any product that may be evaluated in this article, or claim that may be made by its manufacturer, is not guaranteed or endorsed by the publisher.
