# Supplementary material for: The novel bio-SYNTAX scoring system for predicting the prognosis of patients undergoing percutaneous coronary intervention with left main coronary artery disease
Source: Front Cardiovasc Med. 2022 Sep 23;9:912286. doi: 10.3389/fcvm.2022.912286 (PMC9538309; doi:10.3389/fcvm.2022.912286)
Supplement: Supplementary file 1 [file Table_1.DOCX]

Supplementary Table 1. Univariate analysis for major adverse cardiac events

|  | MACE | |  |
| --- | --- | --- | --- |
| Variables | No  (N=195) | Yes  (N=80) | *p* value |
| SS | 23.6±7.2 | 26.7±8.0 | 0.002 |
| SS II | 29.4±9.9 | 36.0±12.8 | <0.001 |
| Bio-CSS | 34.6±18.2 | 51.8±28.3 | <0.001 |
| Age (year) | 63.6±10.3 | 66.7±10.8 | 0.026 |
| Male, n (%) | 139 (71.3%) | 65 (81.2%) | 0.05 |
| Body mass index (Kg/m^2^) | 23.6±2.6 | 23.5±2.5 | 0.891 |
| Clinical presentation |  |  | 0.05 |
| Chronic stable angina, n (%) | 59 (30.3%) | 16 (20.00%) |  |
| Acute coronary syndrome, n (%) | 136 (69.7%) | 64 (80.0%) |  |
| Past history |  |  |  |
| Coronary heart disease, n (%) | 35 (18.6%) | 19 (27.5%) | 0.120 |
| Hypertension, n (%) | 103 (54.8%) | 34 (49.3%) | 0.433 |
| Diabetes mellitus, n (%) | 61 (32.4%) | 26 (37.7%) | 0.432 |
| Hyperlipidemia, n (%) | 61 (32.4%) | 19 (27.5%) | 0.451 |
| Current smoking, n (%) | 109 (58.0%) | 47 (68.1%) | 0.140 |
| Left ventricular ejection fraction (%) | 56.1±9.8 | 47.3±13.1 | <0.001 |
| Serum creatinine (mg/dL) | 0.96±0.64 | 1.47±1.60 | 0.007 |
| Log NT-proBNP (pg/mL) | 5.45±1.52 | 6.51±2.04 | <0.001 |
| Discharge medication |  |  |  |
| Aspirin, n (%) | 167 (88.4%) | 51 (83.60%) | 0.334 |
| Clopidogrel, n (%) | 173 (91.5%) | 55 (90.2%) | 0.743 |
| ACE-I/ARBs, n (%) | 122 (64.6%) | 35 (57.4%) | 0.314 |
| Beta-blockers, n (%) | 169 (89.4%) | 48 (78.7%) | 0.031 |
| Statins, n (%) | 156 (82.5%) | 40 (65.6%) | 0.005 |
| Diuretics, n (%) | 39 (20.6%) | 26 (42.6%) | 0.001 |
| LMCA status |  |  | 0.194 |
| LMCA, isolated, n (%) | 21 (10.8%) | 7 (8.8%) |  |
| LMCA + 1-vessel disease, n (%) | 28 (14.4%) | 9 (11.2%) |  |
| LMCA + 2-vessel disease, n (%) | 47 (24.1%) | 15 (18.8%) |  |
| LMCA + 3-vessel disease, n (%) | 99 (50.8%) | 49 (61.3%) |  |
| LM bifurcation, n (%) | 155 (79.5%) | 67 (83.8%) | 0.416 |
| LM Stent size (mm) | 3.50±0.32 | 3.51±0.56 | 0.868 |
| LM Stent length (mm) | 23.24±6.28 | 22.95±5.84 | 0.730 |
| Reference vessel diameter (mm) | 3.49±0.40 | 3.46±0.48 | 0.638 |
| Minimal lumen diameter (mm) | 1.83±1.71 | 1.81±1.80 | 0.917 |
| Drug-eluting stent type |  |  | 0.474 |
| Sirolimus eluting stent, n (%) | 7 (3.6%) | 3 (3.8%) |  |
| Paclitaxel eluting stent, n (%) | 28 (14.4%) | 17 (21.2%) |  |
| Zotarolimus eluting stent, n (%) | 56 (28.7%) | 19 (23.8%) |  |
| Everolimus eluting stent, n (%) | 87 (44.6%) | 36 (45.0%) |  |
| Biolimus eluting stent, n (%) | 17 (8.7%) | 5 (6.2%) |  |
| LM stenting strategy |  |  | 0.212 |
| 1 stent strategy, n (%) | 176 (90.3%) | 68 (85.0%) |  |
| 2 stent strategy, n (%) | 19 (9.77%) | 12 (15.0%) |  |

Data expressed as mean ± SD or number (percent)

SS = SYNTAX score; SS II = SYNTAX score II; Bio-CSS = Biomarker-Clinical SYNTAX score; NT-proBNP = N-terminal pro-B type natriuretic peptide; ACE-I/ARBs = Angiotensin-converting enzyme inhibitors/angiotensinogen type II receptor blockers; LMCA = left main coronary artery; LM = left main
