# Supplementary figures and images for: The novel bio-SYNTAX scoring system for predicting the prognosis of patients undergoing percutaneous coronary intervention with left main coronary artery disease
Source: Front Cardiovasc Med. 2022 Sep 23;9:912286. doi: 10.3389/fcvm.2022.912286 (PMC9538309; doi:10.3389/fcvm.2022.912286)

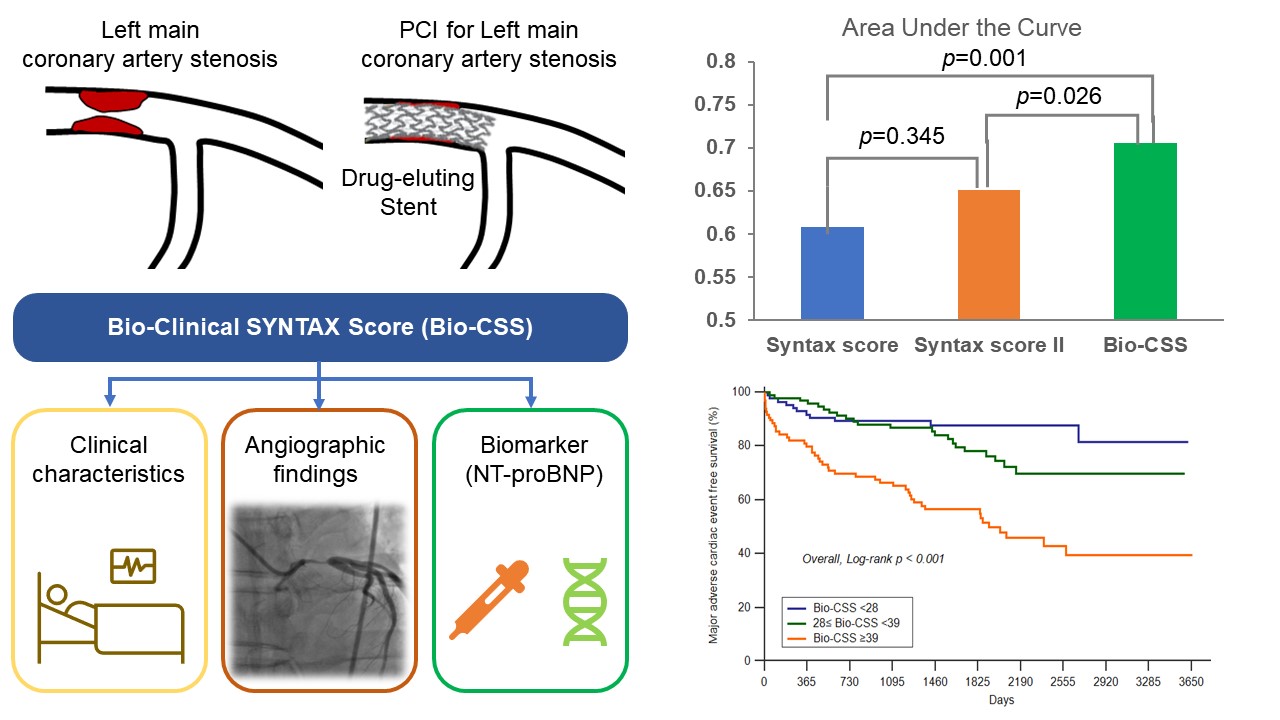

Supplement: Supplementary file 2 [file Image_1.JPEG]
